# Influence of nursing cardiac rehabilitation on cardiac function and quality of life in patients with coronary heart disease after percutaneous coronary intervention

**DOI:** 10.3389/fcvm.2026.1582925

**Published:** 2026-05-15

**Authors:** Shujuan Wu, Ming Ma, Caiting Ning, Yanling Cheng

**Affiliations:** 1 Department of Cardiology, Tianjin Rehabilitation Center of Joint Logistics Support Force, Tianjin, China; 2 Department of Economics and Management Section, Tianjin Rehabilitation Center of Joint Logistics Support Force, Tianjin, China; 3 Department of Special Clinic, Tianjin Rehabilitation Center of Joint Logistics Support Force, Tianjin, China

**Keywords:** cardiac function, coronary heart disease, nursing cardiac rehabilitation, percutaneous coronary intervention, quality of life

## Abstract

**Background:**

With the widespread application of Percutaneous coronary intervention (PCI) is widely used to treat coronary heart disease (CHD). Although it can effectively open narrowed or occluded coronary arteries, patients still face many challenges in terms of postoperative cardiac function recovery and improvement of quality of life. Therefore, this study aimed to evaluate the impact of nursing cardiac rehabilitation on cardiac function and quality of life in patients with CHD after PCI.

**Methods:**

From February 2022 to February 2023, 100 patients with CHD who underwent PCI at the Tianjin Rehabilitation Center of the Joint Logistics Support Force were selected and divided into the control group (CG) and the observation group (OG). The CG received routine nursing care. The OG underwent cardiac rehabilitation nursing.

**Results:**

Compared with the CG, the OG had a lower left ventricular end-systolic diameter and left ventricular end-diastolic diameter and a higher left ventricular ejection fraction after nursing (*P* < 0.05). Compared with the CG, the OG had a longer 6-min walking distance (6MWD) after nursing (*P* < 0.05). The incidence of adverse cardiovascular events was lower in the OG than in the CG (*P* = 0.025). Compared with the CG, the OG had higher World Health Organization Quality of Life-BREF (WHOQOL-BREF) scores in all fields after nursing (*P* < 0.05). Nursing satisfaction was higher in the OG than in the CG (*P* = 0.014).

**Conclusions:**

Nursing cardiac rehabilitation can promote cardiac function and quality of life in patients with CHD after PCI, which has high clinical significance.

## Introduction

Coronary heart disease (CHD) is a common condition in the field of cardiovascular medicine (1). The pathological mechanism arises from the development and progression of atherosclerotic plaques in the coronary arteries due to the long-term influence of various risk factors, such as hypertension, hyperlipidemia, diabetes, and smoking (2). This continuous process of damage can lead to narrowing or occlusion of the coronary artery lumen, resulting in insufficient blood supply to the myocardium, which, in turn, causes myocardial ischemia and hypoxia (2). The clinical manifestations are typical symptoms, such as angina pectoris and chest tightness (3). If the condition progresses to acute coronary artery occlusion, it may trigger acute myocardial infarction, causing irreversible necrosis of cardiac cells and even leading to fatal complications such as malignant arrhythmias and cardiogenic shock (4).

Percutaneous coronary intervention (PCI) has become the core method for revascularization of coronary arteries in patients with CHD (5). This technique rapidly opens diseased vessels through balloon dilation and stent implantation, significantly improving myocardial perfusion levels and effectively reducing the mortality rate of patients with acute myocardial infarction (6). However, patients after PCI continue to face issues such as in-stent restenosis, incomplete recovery of cardiac function, and decreased quality of life (7). Current studies have shown that nursing cardiac rehabilitation, as a systematic intervention model, can significantly improve patients' cardiac function through comprehensive measures such as exercise training, risk factor management, psychological support, and health education (8).

Nursing cardiac rehabilitation is a multidimensional rehabilitation system designed for patients with cardiovascular diseases. Its core objective is to promote recovery of cardiac function, optimize disease management behaviors, and enhance the health-related quality of life through structured intervention (9). According to the international guidelines for cardiovascular rehabilitation, implementing cardiac rehabilitation after PCI can reduce the all-cause mortality rate of patients by 20%–30%, decrease the rehospitalization rate by 25%, and significantly improve their exercise tolerance and mental health status (10). Currently, the implementation rate of nursing cardiac rehabilitation in China is less than 20%, and the intervention plans lack a standardized evaluation system, resulting in significant heterogeneity in the rehabilitation outcomes of patients (11).

Based on this, this study aimed to conduct a randomized controlled trial to systematically evaluate the multidimensional effects of nursing cardiac rehabilitation on the cardiac function and quality of life of patients with CHD after PCI, as well as nursing satisfaction. The research results may provide a scientific basis for optimizing the rehabilitation path after PCI and establishing individualized nursing cardiac rehabilitation plans. This, in turn, aims to achieve the research goal of improving the long-term prognosis of patients and enhancing the efficiency of medical resource utilization.

## Methods

### General data

From February 2022 to February 2023, 100 patients with CHD who underwent PCI at the Tianjin Rehabilitation Center of the Joint Logistics Support Force were selected and randomly divided into the control group (CG) and an observation group (OG), with 50 patients in each group. This study was approved by the Ethics Committee of the Tianjin Rehabilitation Center of the Joint Logistics Support Force. All patients provided informed consent.

The inclusion criteria were as follows: (1) CHD was confirmed using color Doppler ultrasound and electrocardiography. (2) Patients who underwent PCI treatment. (3) Age over >18. The exclusion criteria were as follows: (1) Impaired liver and kidney function. (2) Concurrent heart failure, arrhythmia, and other heart diseases. (3) Patients with malignant tumors. (4) Patients with mental abnormalities or communication disorders who were unable to cooperate in completing the treatment.

### Data collection

Before the patient underwent PCI, baseline data were collected through reviewing medical records and conducting face-to-face interviews with the patient and their family members. The data included age, gender, course of disease, smoking history, drinking history, history of hypertension, history of diabetes, and cardiac function classification (according to the New York Heart Association cardiac function classification standard). All data were collected by researchers who had received unified training using standardized questionnaires to ensure data accuracy and consistency.

### Sample size calculation

Power analysis was carried out using G*Power 3.1.9.7 software to determine the sample size required to detect statistical differences. With an alpha level of 0.05% and a power analysis of 90%, the study revealed that a sample size of 50 patients per group was required.

### Randomization and blinding

A group randomization design was adopted for randomization. A random allocation sequence was generated using a computer. Allocation confidentiality measures were achieved through sequential numbering, sealing, and opaque envelopes. After meeting the inclusion criteria, patients were randomly assigned to the CG or OG in a 1:1 ratio. To further ensure the objectivity and fairness of the research results, strict confidentiality measures were adopted in the result evaluation stage. The personnel responsible for result evaluation were completely independent from those involved in patient grouping, did not participate in the random allocation process of patients, and did not access any information related to patient grouping throughout the entire research process. Prior to the evaluation, specialized training was provided to the personnel. This training emphasized the importance of confidentiality and strictly prohibited them from obtaining any information about patient grouping through any means. At the same time, during the data collection and analysis stage, strict management was implemented for information related to grouping, allowing only specific and authorized researchers to access it. The result evaluation personnel could only obtain the necessary data for evaluating the observation indicators, thereby ensuring that the result evaluation personnel maintained confidentiality regarding the grouping situation.

### Study methods

The CG received routine nursing care. Nurses strengthened postoperative monitoring and carefully observed the patient's heart rate, blood pressure, respiratory rate, and other signs to avoid adverse events. After the patient's condition and signs were stable, the nurses guided the patient to start exercising and gradually increase the intensity of the exercise.

The OG underwent cardiac rehabilitation nursing. The specific processes were as follows:

(1) Health education: Appropriate education methods were selected according to the education level of patients, and relevant knowledge of CHD was carefully popularized through health knowledge lectures, health knowledge manuals, videos, oral education, and other means, emphasizing the positive role of postoperative rehabilitation training in disease rehabilitation and attracting patients' attention.
(2) Emotional counseling: The Self-rating Anxiety Scale (SAS) and the Self-rating Depression Scale (SDS) were adopted for observing and evaluating whether a negative situation occurred in patients. The SDS and SAS, each consisting of 20 items, respectively, used a four-level scoring system. Scores <50 indicate no anxiety or depression, 50–59 indicate mild anxiety or depression, 60–69 indicate moderate anxiety or depression, and >70 indicate severe anxiety/depression. The Cronbach’s reliability coefficient for the scales was adequate (*α* = 0.826 for SDS and *α* = 0.816 for SAS), indicating that the SDS and SAS scales have good reliability and validity (12). If the patient had SDS and SAS scores ≥ 50, the professional psychological counseling nurses provided targeted psychological intervention measures.
(3) Exercise guidance: (1) In the early postoperative period, patients were encouraged to engage in passive exercises, such as maintaining sitting and standing posture, walking slowly, and navigating stairs. Exercise intensity was determined using ECG, myocardial injury markers, exercise cardiopulmonary test, and other results, with exercise prescription provided by doctors and nurse guidance. (2) Between 2 and 5 weeks after surgery, patients were instructed to perform 3–5 moderate-intensity exercises per week, including the following: (a) Aerobic exercises, such as walking, jogging, and swimming for 30 min each session. (b) Balance training involves using balance mats or balancing machines to help reduce the risk of falling. (c) Resistance exercises. The nurses instructed the patients to perform push-ups, dumbbell bends, and other exercises to train their muscle groups. d. Flexibility exercises. After completing the above exercises, the patient's body was stretched for approximately 10 min. (3) After discharge, the patient was informed to maintain an appropriate amount of exercise every day, such as walking, jogging, Taijiquan, square dancing, playing table tennis, etc. Patients should ensure that their exercise intensity is not excessive and should monitor their blood pressure, pulse, and other basic vital signs before engaging in cardiac rehabilitation training, ensuring no abnormalities are present. In the presence of chest pain, chest tightness, shortness of breath, rapid heart rate, sweating, and other discomfort, exercise was immediately suspended, and serious medical treatment was provided.
(4) Dietary guidance, Nutritional dietary plans were tailored to patients based on their taste preferences and eating habits, focusing on foods that are low in salt and fat and easy to digest. Patients needed to increase their intake of nutrients such as protein and vitamins while avoiding raw and cold-stimulating foods to ensure t they received enough nutrients to speed up the body’s recovery.
(5) Medication guidance: Patients were instructed to take anticoagulants, lipid-lowering medications, and other drugs following surgery. The method of use, dosage, and adverse reactions of each drug were explained in detail, and patients were advised to adhere strictly to the doctor's instructions.
(6) Posthospital follow-up: After the patient was discharged, the nurse consistently monitored the patient's recovery through phone calls, WeChat, and other methods. The nurse corrected the patient's any detrimental behaviors and advised the patient to visit the hospital for regular check-ups.

### Observation indicators

(1) Cardiac function: Left ventricular end-diastolic diameter (LVEDD), left ventricular end-systolic diameter (LVESD), and left ventricular ejection fraction (LVEF) were measured using ultrasonography before nursing and at discharge.
(2) Exercise endurance: The 6MWD of patients in both groups was compared (13). The 6MWD was measured using a 50-m section of a 60-m-long corridor in the patient's ward area. The nurses marked every 3 m, and instructed the patient to walk back and forth at their fastest pace for 6 min. The distance covered during the walk was measured before nursing and at discharge.
(3) The incidence of adverse cardiovascular events, such as arrhythmia, myocardial infarction, and angina, was compared between the two groups at discharge.
(4) The World Health Organization Quality of Life-BREF (WHOQOL-BREF) scores were used to evaluate the quality of life of patients in both groups before nursing and at discharge. The reliability of the WHOQOL-BREF domains and the overall quality of life were assessed using Cronbach’s *α* coefficient, with scores of 0.70 and above deemed acceptable (14). It consisted of 26 assessment items, which evaluated four aspects: psychological, environmental, and social relation fields. The scores for each aspect ranged from 0 to 100 points. A higher score indicates a higher quality of life.
(5) A self-made nursing satisfaction scale was used to investigate patient satisfaction at discharge. The Cronbach *α* coefficient of the total internal consistency reliability of the questionnaire was 0.846, indicating a good evaluation effect. Satisfaction levels were divided according to the patient scores: very satisfied (>90 points), generally satisfied (70–90 points), and dissatisfied (<70 points). Total nursing satisfaction = very satisfied rate + satisfaction rate.

### Statistical analysis

The data were processed using SPSS 23.0 statistical software. All continuous variables are presented as mean ± standard deviation (*x* ± *s*) and were tested for normality using the Shapiro–Wilk test. Normally distributed variables were compared using Student's *t*-test, while non-normally distributed variables were compared using the Mann–Whitney *U* test. Categorical variables were represented by [*n* (%)], and the *χ*^2^ test was performed for comparison between the two groups. To account for multiple comparisons across the four domains of the WHOQOL-BREF, the Bonferroni correction was used, with a corrected significance level set at *α* = 0.05/4 = 0.0125. *P* < 0.05 indicated that the difference was statistically significant, except where a stricter threshold was specified for multiple comparisons.

## Results

### General data of patients between the two groups

There were no significant differences in gender, age, course of disease, smoking history, drinking history, history of hypertension, history of diabetes, or cardiac function classification in the general data between the two groups (*P* > 0.05, Table 1), indicating comparability.

**Table 1:** General data of patients in both groups.

Items	Control group (*n* = 50)	Observation group (*n* = 50)	*χ*^2^/t	P
Gender			0.040	0.840
Male	28 (56.00)	27 (54.00)		
Female	22 (44.00)	23 (46.00)		
Age (years)	59.85 ± 6.34	60.04 ± 6.38	0.149	0.881
Course of disease (years)	3.12 ± 0.83	3.18 ± 0.85	0.357	0.721
Smoking history	23 (46.00)	25 (50.00)	0.160	0.688
Drinking history	20 (40.00)	22 (44.00)	0.164	0.685
History of hypertension	18 (36.00)	21 (42.00)	0.378	0.538
History of diabetes	15 (30.00)	17 (34.00)	0.183	0.668
Cardiac function classification			0.332	0.846
Grade I	24 (48.00)	23 (46.00)		
Grade II	20 (40.00)	19 (38.00)		
Grade III	6 (12.00)	8 (16.00)		

### Cardiac function in both groups

**Figure 1:**
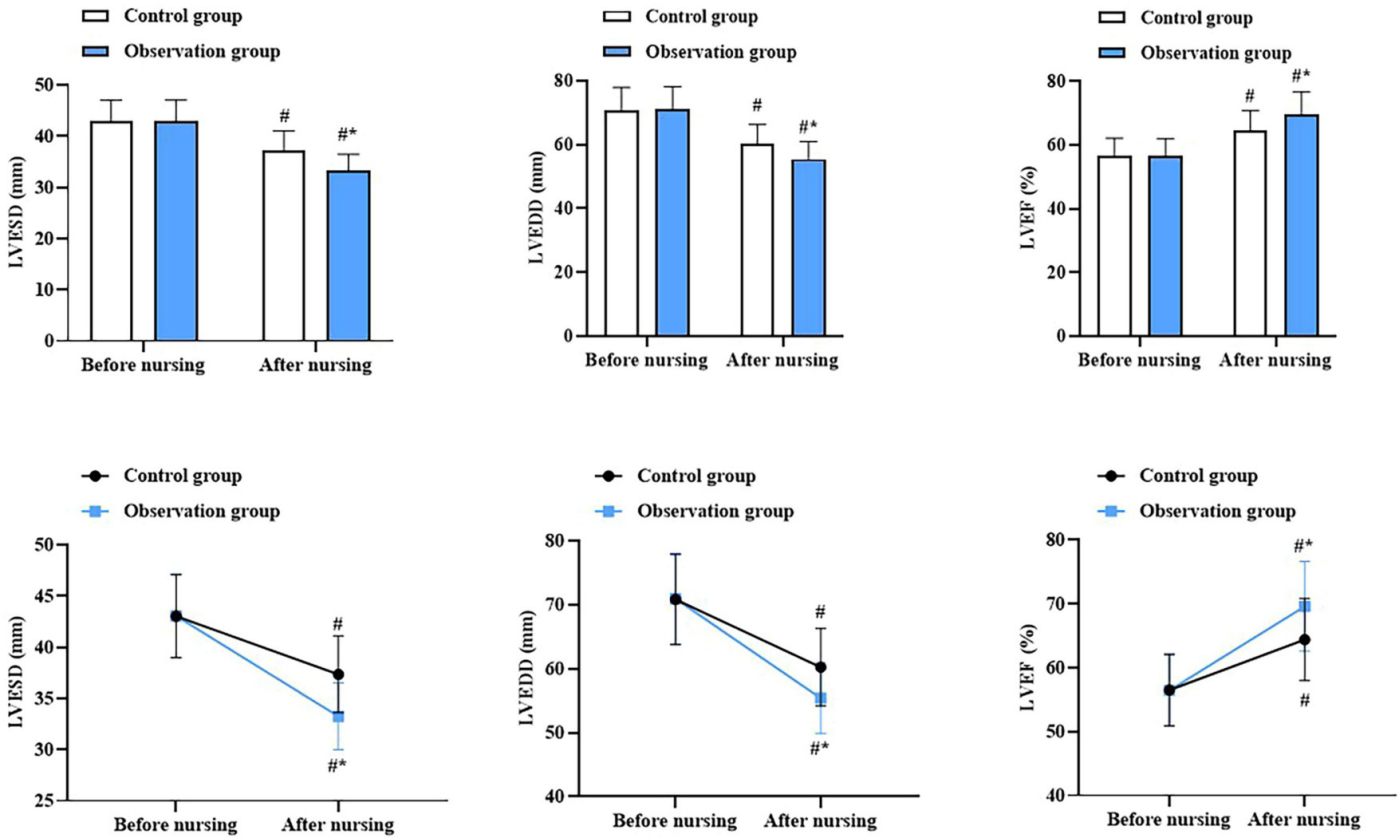
Cardiac function in both groups. ^#^*P* < 0.05, compared with before nursing. *P < 0.05, compared with CG.

Before nursing, there was no difference in cardiac function indicators between the two groups (*P* > 0.05). At discharge, LVESD and LVEDD decreased in both groups, while the LVEF increased (*P* < 0.05). More importantly, compared with the CG, the OG had lower LVESD and LVEDD and higher LVEF at discharge (*P* < 0.05, Figure 1).

### Exercise endurance in both groups

**Figure 2:**
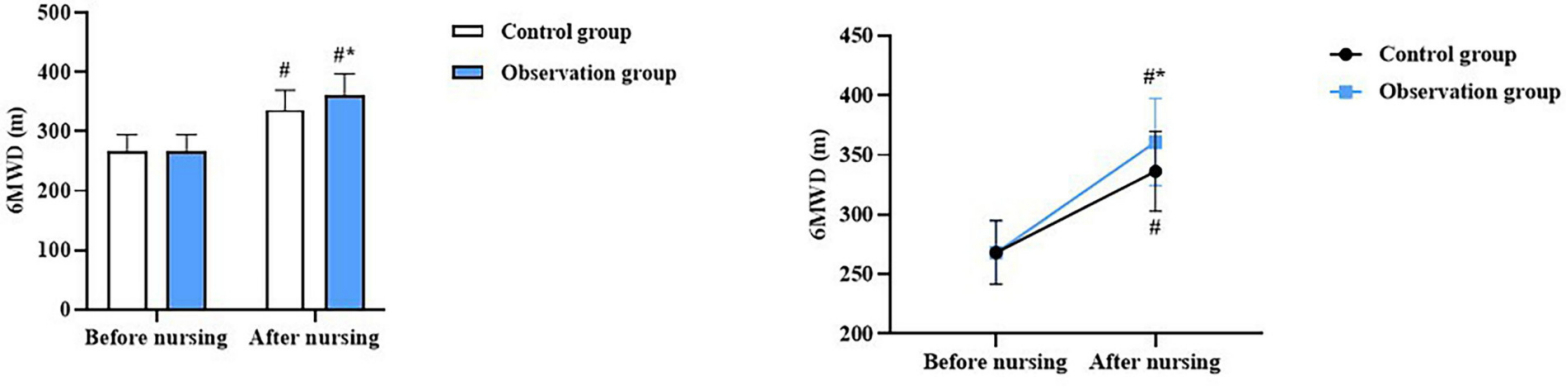
Exercise endurance in both groups. ^#^*P* < 0.05, compared with before nursing. *P < 0.05, compared with CG.

Before nursing, there was no difference in the 6MWD between the two groups (*P* > 0.05). At discharge, the 6MWD increased in both groups, with the OG showing a higher increase than the CG (*P* < 0.05, Figure 2).

### Incidence of adverse cardiovascular events in both groups

In the CG, three patients experienced arrhythmia, three patients experienced myocardial infarction, and three patients experienced angina. The overall incidence rate was 18% (9/50). In the OG, one patient experienced arrhythmia, and one patient experienced angina. The overall nursing satisfaction was 4% (2/50). The incidence of adverse cardiovascular events in the OG was lower than that in the CG (*P* = 0.025, Table 2).

**Table 2:** Incidence of adverse cardiovascular events in both groups.

Groups	Cases	Arrhythmia	Myocardial infarction	Angina	Total incidence rate
Control group	50	3 (6.00)	3 (6.00)	3 (6.00)	9 (18.00)
Observation group	50	1 (2.00)	0 (0.00)	1 (2.00)	2 (4.00)
*χ* ^2^					5.005
*P*					0.025

### Quality of life in both groups

**Figure 3:**
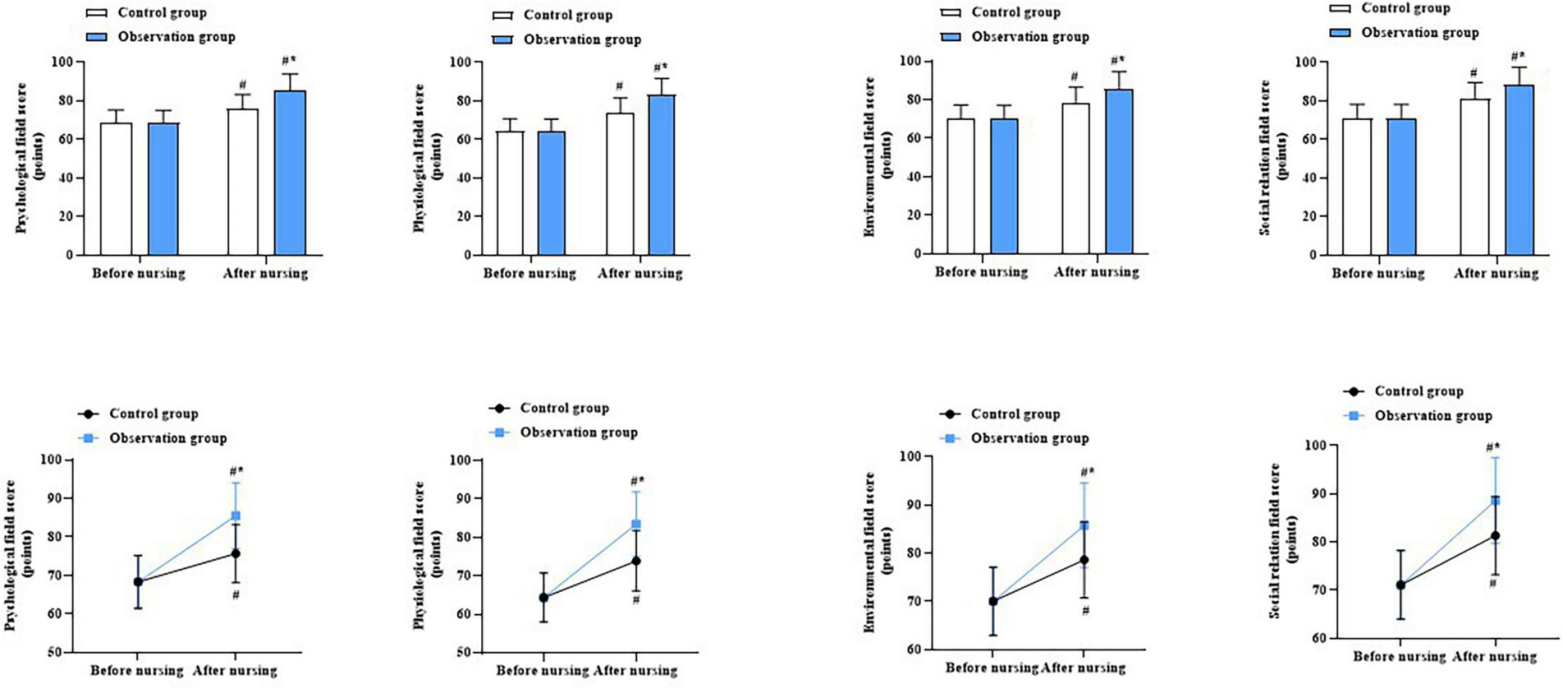
Quality of life in both groups. ^#^*P* < 0.05, compared with before nursing. *P < 0.05, compared with CG.

Before nursing, there were no differences in the WHOQOL-BREF scores across all fields between the two groups (*P* > 0.05). At discharge, the WHOQOL-BREF scores increased in both groups across all fields. After applying the Bonferroni correction (*α* = 0.0125), the OG had significantly higher scores than the CG in all four domains: physiological (*P* = 0.008), psychological (*P* = 0.006), environmental (*P* = 0.009), and social relationship domains (*P* = 0.010) (Figure 3).

### Nursing satisfaction in both groups

In the CG, 23 patients were very satisfied, 17 patients were satisfied, and 10 patients were dissatisfied. The total nursing satisfaction was 80% (40/50). In the OG, 26 patients were very satisfied, 22 patients were satisfied, and four patients were dissatisfied. The total nursing satisfaction was 96% (48/50). The nursing satisfaction of patients in the OG was higher than that in the CG (*P* = 0.014, Table 3).

**Table 3:** Nursing satisfaction in both groups.

Groups	Cases	Very satisfied	Satisfied	Dissatisfied	Total satisfaction rate
Control group	50	23 (46.00)	17 (34.00)	10 (20.00)	40 (80.00)
Observation group	50	26 (52.00)	22 (44.00)	2 (4.00)	48 (96.00)
*χ* ^2^					6.061
*P*					0.014

## Discussion

The common symptoms of CHD are chest pain and shortness of breath, and it may also include symptoms such as vomiting and sweating (15). When the disease worsens, it can trigger a variety of complications that directly affect the normal life of patients and reduce their quality of life (16). PCI is a commonly used treatment method in clinical practice and the main method for patients with CHD to restore blood vessels (17). It can improve the blood supply to the heart muscle and keep the blood vessels unobstructed, thereby alleviating the clinical symptoms of patients and accelerating the recovery of heart function (18). However, most patients remain bedridden after surgery, experiencing poor recovery of cardiac function, which hinders effective improvement in their quality of life. Moreover, they may also suffer from various complications (19). Therefore, effective nursing is necessary for patients after PCI.

In the past, most patients with CHD after PCI received routine nursing care. Although this nursing method had certain effects, it only focused on basic nursing and overlooked guidance for postoperative cardiac function rehabilitation, thereby failing to achieve the expected nursing outcome (20). Nursing cardiac rehabilitation is a recently developed nursing model. It follows the “patient-centered” nursing concept and provides scientific nursing plans based on the actual situation of patients to meet the nursing needs of patients as much as possible (21). Compared with routine nursing, cardiac rehabilitation has a complete rehabilitation management plan. Through a series of measures, such as health education, emotional counseling, exercise guidance, diet guidance, medication guidance, and follow-up, the prognosis of patients can be improved, and their early recovery can be promoted (22).

The core contribution of this study is to systematically validate, through a rigorous randomized controlled trial in the Chinese clinical context, the multidimensional short-term efficacy of a nurse-led cardiac rehabilitation program integrating the five core components (exercise, nutrition, psychology, medication, and follow-up) in patients after PCI. Given the low implementation rate of cardiac rehabilitation nursing in China (<20%), this study provides a practical and replicable intervention protocol for clinical nursing. In our study, the results revealed that compared with the CG, the OG had lower LVESD and LVEDD and higher LVEF at discharge, suggesting that nursing cardiac rehabilitation could promote the cardiac function of patients with CHD after PCI. In addition, compared with the CG, the OG had a longer 6MWD after nursing, suggesting that nursing cardiac rehabilitation could promote exercise endurance in patients with CHD after PCI. This was because, within the nursing cardiac rehabilitation plan, nurses developed exercise regimens tailored to each patient's condition and encouraged them to consistently engage in rehabilitation training. This could enhance the patient's heart function and improve their exercise endurance levels. Consistently, Guan et al. indicated that individualized cardiac rehabilitation can promote cardiac function and the 6MWD in patients undergoing coronary artery bypass grafting (23).

Our study also indicated that after nursing, the incidence of adverse cardiovascular events was lower in the OG compared to that in the CG. In addition, the WHOQOL-BREF scores at discharge were higher in the OG than in the CG, and patient satisfaction with nursing s was higher in the OG than in the CG. These results suggest that nursing cardiac rehabilitation can decrease the incidence of adverse cardiovascular events, improve the quality of life, and promote nursing satisfaction in patients with CHD after PCI. During nursing cardiac rehabilitation, nurses provided dietary guidance to help patients consume more nutritious foods. This enhanced the patients' immunity, further consolidated the effects of surgical treatment, reduced the occurrence of cardiovascular adverse events, and facilitated quicker recover. In accordance with our findings, Arjunan et al. indicated that providing cardiac rehabilitation to patients with heart failure could promote their quality of life(24).

It is important to note that the intervention in this study did not include cardiopulmonary exercise testing (CPET), so no comparisons or claims of superiority over CPET-based assessment can be made. The personalized exercise guidance in this study was based on electrocardiograms, myocardial injury markers, and patients' self-reported tolerance. Future studies should incorporate CPET to provide more objective and individualized exercise prescriptions.

## Conclusion

Nursing cardiac rehabilitation can promote cardiac function and quality of life in patients with CHD after PCI, which has high clinical significance.

## Data Availability

The original contributions presented in this study are included in the article/Supplementary Material, further inquiries can be directed to the corresponding author.
